# Cetuximab-modified mesoporous silica nano-medicine specifically targets EGFR-mutant lung cancer and overcomes drug resistance

**DOI:** 10.1038/srep25468

**Published:** 2016-05-06

**Authors:** Yuetong Wang, Hsin-Yi Huang, Liu Yang, Zhanxia Zhang, Hongbin Ji

**Affiliations:** 1 Key Laboratory of Systems Biology, Institute of Biochemistry and Cell Biology, Shanghai Institutes for Biological Sciences, Chinese Academy of Science, Shanghai, 200031, China; 2 CAS center for Excellence in Molecular Cell Science, Institute of Biochemistry and Cell Biology, Shanghai Institutes for Biological Sciences, Chinese Academy of Science, Shanghai, 200031, China; 3 Innovation Center for Cell Signaling Network, Institute of Biochemistry and Cell Biology, Shanghai Institutes for Biological Sciences, Chinese Academy of Science, Shanghai, 200031, China; 4 School of Life Science and Technology, Shanghai Tech University, Shanghai, 200120, China

## Abstract

Drug resistance to tyrosine kinase inhibitor (TKI) is the main obstacle for efficient treatment of epidermal growth factor receptor (EGFR)-mutant lung cancer patients. Here we design a cetuximab-capped mesoporous silica nanoparticle (MP-SiO_2_ NP) as the drug carrier to specifically target EGFR-mutant lung cancer cells and efficiently release loaded drugs including doxorubicin and gefitinib. This innovative nano-medicine can specifically target lung cancer cells with high EGFR expression rather than those with low EGFR level. Treatment of a gefitinib-resistant cell line derived from PC9 cell (PC9-DR) with the gefitinib-loaded cetuximab-capped MP-SiO_2_ NP showed a significant inhibition of cell growth. Moreover, this nano-medicine successfully suppressed the progression of PC9-DR xenograft tumors. This tumor suppression was due to the endocytosis of large amount of nano-medicine and the effective gefitinib release induced by high glutathione (GSH) level in PC9-DR cells. Collectively, our study provides a novel approach to overcome EGFR-TKI resistance using cetuximab modified MP-SiO_2_ NP, which holds strong potential for effective management of EGFR-mutant lung cancer.

Lung cancer is the leading cause of cancer-related death worldwide^1^. Extensive studies have identified a number of oncogenic driver mutations which can serve as therapeutic targets. One of the most successful examples is the kinase domain mutants of epidermal growth factor receptor (EGFR)^2^. Previous reports demonstrated that deregulation of EGFR was frequently associated with non-small cell lung cancer (NSCLC)^3^,^4^. There are mainly two categories of targeted drugs for EGFR. One is EGFR-targeted tyrosine kinase inhibitors (TKIs), including gefitinib (GEF) and erlotinib. The other is the anti-EGFR monoclonal antibody, such as cetuximab (CET) and panitumumab^5^,^6^. However, drug resistance to these therapeutic reagents is the main obstacle to the successful targeted therapy in clinic^7^,^8^.

In recent years, the mesoporous SiO_2_ nanoparticle (MP-SiO_2_ NP) attracts substantial interest due to its unique properties, such as high drug-loading capability from their large surface area and pore volume, facile tuning of the particle size over a broad range, specific targeting through modifying or bioconjugating the particle surface, and high biochemical and physicochemical stability^9^. These properties of MP-SiO_2_ NP were implemented to develop new drug delivery systems^10^,^11^, catalysts^12^,^13^ and imaging materials^14^,^15^. Specifically, the capping of the pores which include entrapped substrates with stimuli-sensitive units enables the gating of the pores by the signal-triggered “unlocking”, and the controlled-release of the entrapped substrates. Different stimulus, such as pH^16^,^17^, redox reagents^18^,^19^,^20^, photonic signals^21^,^22^, and enzymes^23^,^24^ were established as the triggers to unlock the functional gates. Recently, the stimuli like glutathione (GSH), was implemented to unlock the pores via cleaving the disulfide bonds^25^. For example, cyclodextrin-gated, polyethylene glycol-coated MP-SiO_2_ NP exhibited an efficient GSH-mediated doxorubicin (DOX) release in cancer cells^26^. Moreover, it was also reported that the capping with the EGFR antibody CET resulted in specific targeting to cancer cells with high EGFR level^27^. Similarly, another report showed gold nanoparticle coated with CET can target to pancreatic adenocarcinoma with EGFR overexpression^28^.

Here, we developed the cetuximab-capped MP-SiO_2_ NP as the drug carrier to specifically target EGFR-mutant lung cancer cells and efficiently release loaded drugs including doxorubicin and gefitinib. Our data showed that this modified nano-medicine can overcome EGFR-TKI resistance and holds therapeutic implication for effective management of EGFR-mutant lung cancer.

## Results and Discussion

**Figure 1:**
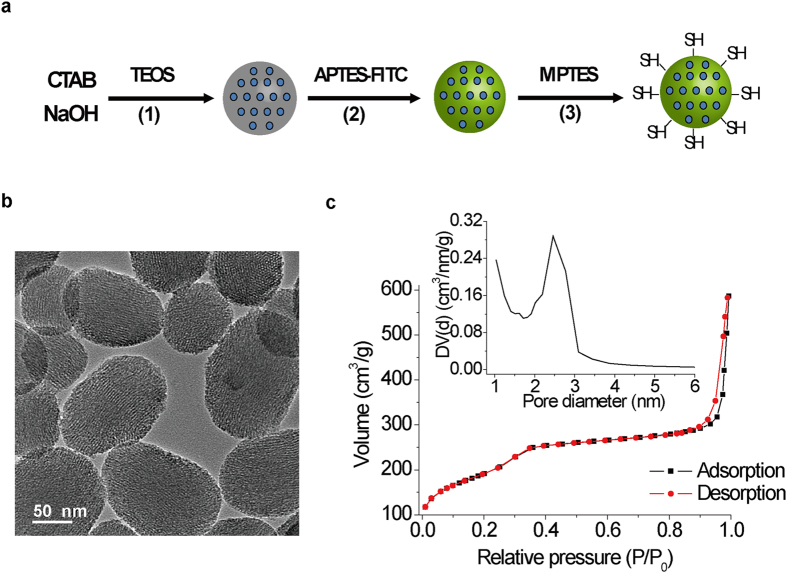
Synthesis and characterization of spherical mesoporous SiO_2_ nanoparticles (MP-SiO_2_ NP). **(a)** Synthesis process of fluorescein isothiocyanate (FITC)-labeled and mercapto-functionalized MP-SiO_2_ NP. **(b)** Transmission electron microscopy (TEM) image of synthesized MP-SiO_2_ NP. The diameter is about 100 nm, scale bar: 50 nm. **(c)** N_2_ adsorption-desorption isotherms of MP-SiO_2_ NP. The specific surface area (BET analysis) is 887.9 m^2^/g. The pore size (BJH analysis) is 2.5 nm.

First, we synthesized the MP-SiO_2_ NP according to previous report^29^. To trace the intracellular MP-SiO_2_ NP, we labeled these nano-particles with fluorescein isothiocyanate (FITC). The surface of the MP-SiO_2_ NP was functionalized with 3-mercaptopropyltriethoxysilane (MPTES) to introduce the mercapto-groups (Fig. 1a). High resolution transmitting electronic microscopy (HRTEM) image showed that the size of spherical MP-SiO_2_ NP was about 100 nm, and the channels of the MP-SiO_2_ NP were well-organized (Fig. 1b). Nitrogen adsorption-desorption isotherms indicated that the MP-SiO_2_ NP possessed relatively high specific surface area (887.9 m^2^/g), well-defined pore size (2.5 nm), and appropriate pore volume (0.92 cm^3^/g) (Fig. 1c).

**Figure 2:**
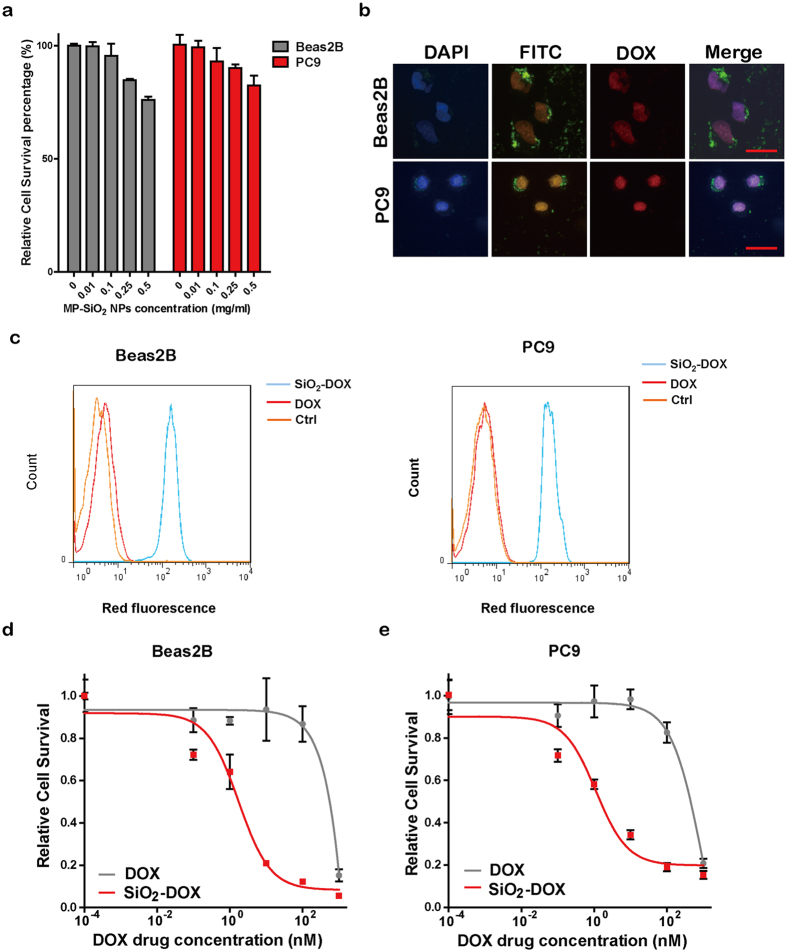
Biocompatibility and drug delivery of MP-SiO_2_ NP. (**a)** Toxicity testing. Relative cell survival of Beas2B and PC9 cells with and without MP-SiO_2_ NP treatments. Bars represent mean ± SEM (n = 4). **(b)** Microscopic images of Beas2B and PC9 cells incubated with 0.01 mg/ml of DOX-loaded MP-SiO_2_ NP for 4 hours (blue: DAPI; green: FITC; red: DOX). Scale bar: 50 μm. **(c)** FACS analyses of DOX uptake in Beas2B and PC9 cells with indicated treatments. **(d)** Relative cell survival of Beas2B cells treated with either free DOX or DOX-loaded MP-SiO_2_ NP. Bars represent mean ± SEM (n = 4). **(e)** Relative cell survival of PC9 cells treated with free DOX or DOX-loaded MP-SiO_2_ NP. Bars represent mean ± SEM (n = 4).

To assess the potential application of MP-SiO_2_ NP, the toxicity of MP-SiO_2_ NP was examined in Beas2B (an immortalized human normal lung epithelial cell line) and PC9 (a human EGFR-mutant lung cancer cell line) cells. As depicted in Fig. 2a, MP-SiO_2_ NP showed toxicity to Beas2B and PC9 cells only in a concentration higher than 0.5 mg/ml. Therefore, we used the MP-SiO_2_ NP at a concentration lower than 0.5 mg/ml for further studies. We characterized the efficacy of MP-SiO_2_ NP’s endocytosis in both cell lines. As shown in Fig. 2b, the fluorescence of FITC (from MP-SiO_2_ NP) were observed in the cytoplasm of both Beas2B and PC9 cells, demonstrating that the MP-SiO_2_ NP had the capability to enter cells through endocytosis. We then loaded the chemotherapeutic agent DOX, which had intrinsic red fluorescence, to MP-SiO_2_ NP. We observed red fluorescence in the nucleus of both cells treated with DOX-loaded MP-SiO_2_ NP through microscope. Through flow cytometry analyses, we detected stronger signal for the red fluorescence in cells treated with DOX-loaded MP-SiO_2_ NP in contrast to free DOX (Fig. 2c).

We further investigated the viability of Beas2B and PC9 cells treated with free DOX or DOX-loaded MP-SiO_2_ NP. Treatment of DOX-loaded MP-SiO_2_ NP resulted in a significantly higher inhibition of both Beas2B and PC9 cell proliferation than free DOX, despite of comparable DOX concentration (Fig. 2d,e). These results suggested that the MP-SiO_2_ NP had a higher drug delivery capability and DOX-loaded MP-SiO_2_ NP possessed the better therapeutic effect. This might be due to the different ways for these reagents to enter cells, e.g., MP-SiO_2_ NP entered cells via endocytosis whereas free DOX through diffusion. In this aspect, endocytosis seemed more efficient for carrying high amount of DOX in contrast to simple diffusion through cell membrane^30^,^31^. Together, these data demonstrated that the MP-SiO_2_ NP is a safe and efficient drug-carrier for treatment.

**Figure 3:**
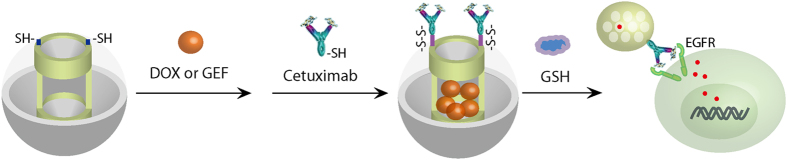
Synthesis of drug-loaded CET-capped MP-SiO_2_ NP. Schemetic illustration of the synthesis of the DOX- or GEF-loaded, MP-SiO_2_ NP with CET capping.

Based on this, we further designed a nano-medicine using the GSH to mediate the drug release (Fig. 3). We first loaded mercapto-functionalized MP-SiO_2_ NP with the therapeutic reagents including DOX and GEF. We then capped the channels of MP-SiO_2_ NP with the EGFR antibody CET as targeting agent NP through the cross-linking of disulfide bond. We estimated that the amount of assembled CET was approximate 1.6 mg/ml. Through the binding of CET to EGFR on cell surface, MP-SiO_2_ NP could be endocytosed and the capped drugs could be released through the cleavage of disulfide bond via the interaction with high GSH level in cytoplasm of cancer cells.

**Figure 4:**
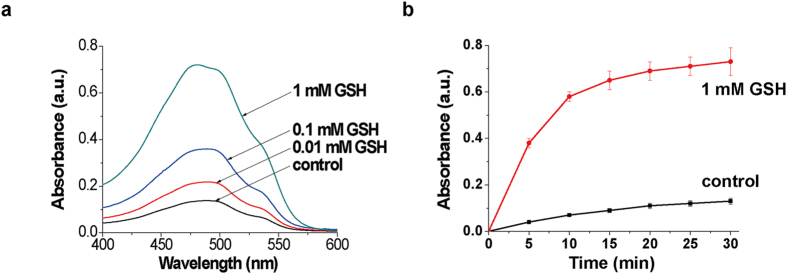
GSH stimulated the drug release from DOX-loaded CET-capped MP-SiO_2_ NP. (**a)** Absorbance spectra corresponding to the released DOX from CET-capped MP-SiO_2_ NP treated with different concentrations of GSH for 1 hr. **(b)** Time-dependent absorbance changes observed upon the DOX release from CET-capped MP-SiO_2_ NP without and with 1 mM GSH. Bars represent mean ± SEM (n = 3).

Indeed, our data showed that the release of DOX from the pores of MP-SiO_2_ NP could be controlled by GSH (Fig. 4a). As the concentration of GSH increased, the absorbance spectra of the released DOX were intensified (Fig. 4a). In the presence of 1 mM GSH, the absorbance intensity increased with time, and reached saturation point after about 30 minutes (Fig. 4b). From the saturated absorbance intensity observed from the GSH-triggered release of DOX and the extinction coefficience of DOX (14700 L·mol^−1^·cm^−1^), we estimated that the amount of released DOX was about 34.7 μM. However, in the absence of GSH, a small amount of DOX was released from the MP-SiO_2_ NP (Fig. 4a,b). This might be resulted from the leakage of DOX from imperfectly-blocked channels of MP-SiO_2_ NP. Similar results were observed when DTT was used as the stimuli to unlock the pores of MP-SiO_2_ NP (Figure S1a,b). Moreover, when cells were pre-treated with glutathione reduced ethyl ester (GSH OEt) which could increase the GSH level in cells^26^, the DOX release significantly increased (Figure S1c). Conversely, the DOX release decreased when cells were pre-treated with diethyl maleate (DM) which scavenged endogenous GSH^32^ (Figure S1c). Without CET capping, the DOX release showed no response to the changes of GSH levels (Figure S1d). These results demonstrated that the redox triggers, such as GSH or DTT, can stimulate the release of therapeutic reagents from the channels of nano-medicine.

**Figure 5:**
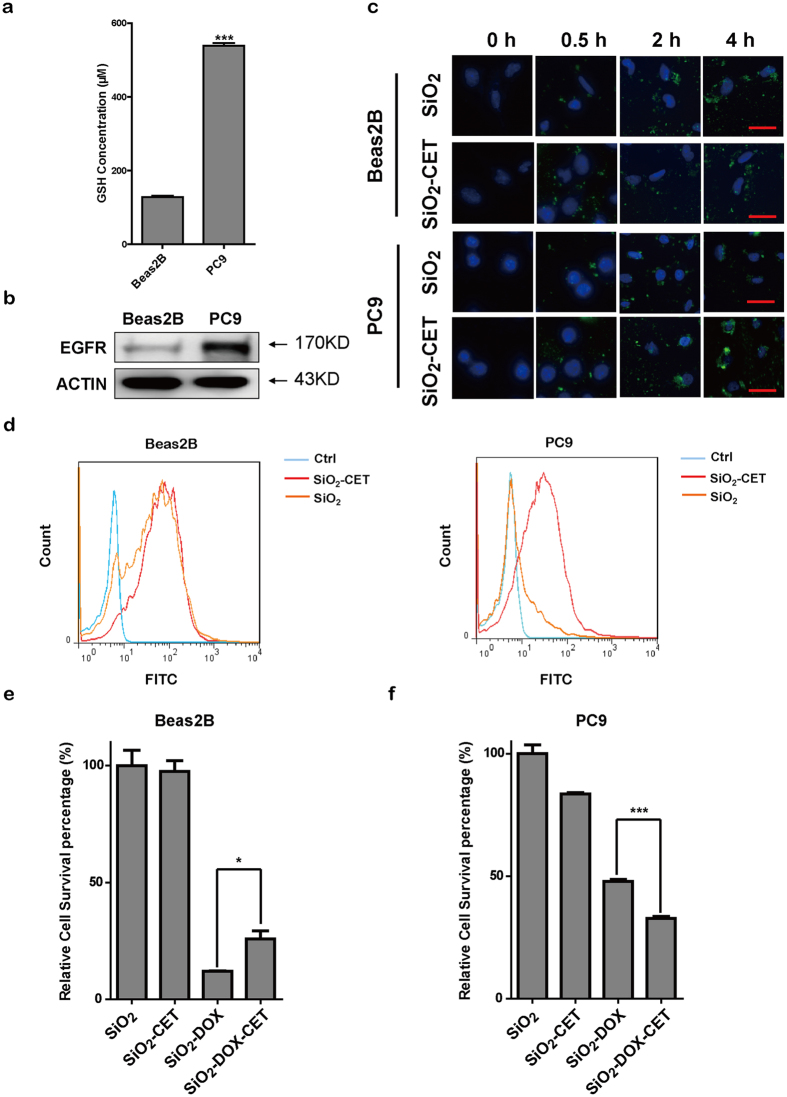
Drug delivery of DOX-loaded CET-capped MP-SiO_2_ NP *in vitro*. **(a)** The cellular GSH levels in Beas2B and PC9 cells. Bars represent mean ± SEM (n = 3). ***p < 0.001. **(b)** Western blot analysis of EGFR expression in Beas2B and PC9 cells. ACTIN served as an internal control. **(c)** Microscopic images of Beas2B and PC9 cells treated with 0.01 mg/ml MP-SiO_2_ NP or CET-capped MP-SiO_2_ NP (blue: DAPI; green: FITC). Scale bar: 50 μm. **(d)** FACS analyses of DOX release in Beas2B and PC9 cells with indicated treatments. **(e)** Relative survival of Beas2B cells treated with MP-SiO_2_ NP, CET-modified MP-SiO_2_ NP, DOX-loaded MP-SiO_2_ NP or DOX-loaded CET-capped MP-SiO_2_ NP for 24 hours. Bars represent mean ± SEM (n = 4). *p < 0.05. **(f)** Relative survival of PC9 cells treated with 0.01 mg/ml of indicated nanoparticles for 24 hours. Bars represent mean ± SEM (n = 4). ***p < 0.001.

We then detected the GSH level and EGFR expression in Beas2B and PC9 cells. As shown in Fig. 5a, the GSH concentration of PC9 was significantly higher (550 μM) than that of Beas2B (150 μM). Also, PC9 cells had a much higher EGFR expression than Beas2B cells (Fig. 5b). In order to verify the specific targeting mediated by CET, both MP-SiO_2_ NP and CET-capped MP-SiO_2_ NP were co-incubated with Beas2B and PC9 cells at different time intervals for endocytosis analyses. A significant difference of FITC fluorescence between these two particles was only observed in PC9 cells but not Beas2B cells, e.g., PC9 cells showed higher FITC fluorescence when with CET-capped MP-SiO_2_ NP in comparison with MP-SiO_2_ NP (Fig. 5c). Flow cytometry analyses demonstrated that treatment of CET-capped MP-SiO_2_ NP resulted in a higher endocytosis than MP-SiO_2_ NP in PC9 cells, whereas in Beas2B cells with low EGFR surface expression no endocytosis difference was observed (Fig. 5d).

We further evaluated the growth inhibition of these nano-medicines in Beas2B and PC9 cells. We found no difference between CET-capped MP-SiO_2_ NP and MP-SiO_2_ NP in inhibiting Beas2B cell proliferation (Fig. 5e,f), indicating that CET had no targeting effect in cells with low EGFR expression. CET-capped MP-SiO_2_ NP was even less efficient than MP-SiO_2_ NP in inhibiting Beas2B cell growth. This might be due to the quick release of DOX from the pores of MP-SiO_2_ NP whereas the cap of CET might sustain the release of therapeutic agent. In PC9 cells, the CET-capped MP-SiO_2_ NP treatment showed a significantly higher growth inhibition than MP-SiO_2_ NP, indicating the nano-medicine with the modification of CET had a great potential and specificity for targeting cancer cells with high EGFR expression.

**Figure 6:**
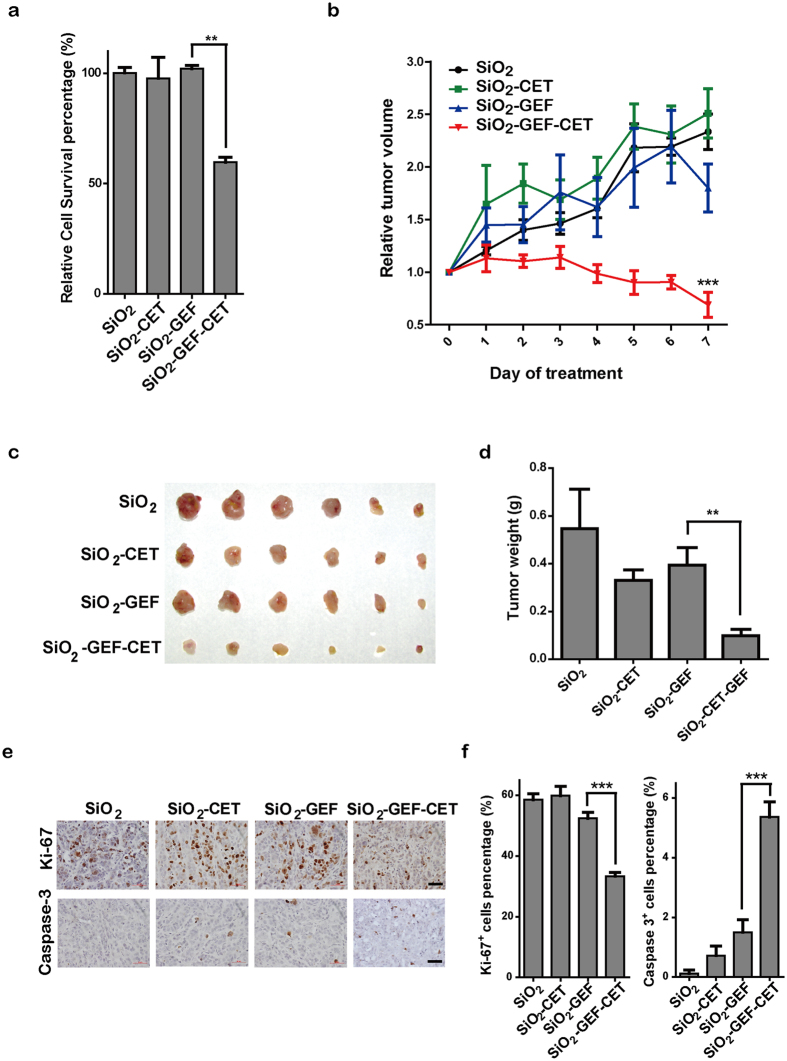
GEF-loaded CET-capped MP-SiO_2_ NP overcame drug resistance *in vitro* and *in vivo*. (**a)** Relative cell survival of PC9-DR cells treated with 0.01 mg/ml of indicated nanoparticles for 24 hours. **(b)** Relative PC9-DR tumor growth of nude mice treated with 50 μl (10 mg/ml) of indicated nanoparticles daily. Bars represent mean ± SD (n = 6). ***p < 0.001. **(c)** Photograph and **(d)** Weights of xenograft tumors from different treatment groups. Bars represent mean ± SD (n = 6). *p < 0.05. **(e)** Ki-67 and caspase-3 immunohistochemical images of PC9-DR tumors after the treatment with indicated nanoparticles. Scale bar: 50 μm. **(f)** Statistic analyses of the percentage of cells positive for Ki-67 and caspase-3 staining. Bars represent mean ± SD (n = 6). ***p < 0.001.

DOX, acting as a cancer-chemotherapeutic agent, has no specificity to EGFR-mutant lung cancer cells and frequently displays toxicity. GEF, one of EGFR-TKIs, attracts more attention due to its good clinical efficacy in EGFR-mutant lung cancer patients. However, patients frequently develop drug resistance and eventually relapse after 6 to 12 months of TKI treatments. We then studied whether the nano-medicine, GEF-loaded CET-capped MP-SiO_2_ NP, had a potential to overcome TKI resistance. For this, we generated the drug-resistant PC9 (PC9-DR) cells through continuous treatment of GEF for about 3 months. As shown in Figure S2a,b, PC9-DR cells were resistant to GEF *in vitro* and *in vivo*. Further study illustrated that PC9-DR cells expressed comparable EGFR level to parental PC9 cells (Figure S2c). Although PC9-DR cells showed significant resistance to GEF treatment, knockdown of EGFR in these cells resulted in a significant inhibition of cell proliferation and survival (Figure S2d–f). We further found that the PC9-DR cells had a higher GSH level than parental PC9 cells, which indicated high GEF release from the GEF-loaded CET-capped MP-SiO_2_ NP in PC9-DR cells (Figure S2g). The CET modification clearly improved the endocytosis of MP-SiO_2_ NP in cells with high EGFR expression (Figure S3a). We further examined the viability of PC9-DR cells after treatments with different MP-SiO_2_ NP. As shown in Fig. 6a, GEF-loaded CET-capped nano-medicine showed the most significant inhibition of the PC9-DR cell growth.

We further explored the potential application of this nano-medicine in PC9-DR tumor treatment *in vivo*. PC9-DR cells were injected subcutaneously into nude mice, and different nano-medicines were then given to mice via *in situ* injection. As shown in Fig. 6b–d, only GEF-loaded CET-capped MP-SiO_2_ NP showed a significant effect upon inhibiting PC9-DR xenograft tumor growth. No obvious loss of body weights was observed (Figure S3b). The immunohistochemical staining of Ki-67 and caspase-3 showed the impressive therapeutic effects of the GEF-loaded CET-capped MP-SiO_2_ NP on PC9-DR xenograft tumors (Fig. 6e,f).

## Conclusion

In this study, we develop a novel mesoporous silica nano-medicine with specific EGFR targeting for overcoming TKI resistance. We synthesize the MP-SiO_2_ NP as an efficient drug carrier which enters cells through endocytosis. We further modify the MP-SiO_2_ NP through loading of either chemotherapeutic agent DOX. We find that DOX-loaded MP-SiO_2_ NP has a higher drug delivery capability and possesses a better therapeutic effect in cancer cells. Based on the modulation of cellular redox level, we design a smart nano-medicine which controls the release of therapeutic reagents from the channels of the MP-SiO_2_ NP through GSH stimulation. Through the specific targeting using the EGFR antibody CET to the channels of MP-SiO_2_ NP, we have established a nano-medicine specifically targeting cancer cells with high EGFR expression. Our data show that the CET-capped MP-SiO_2_ NP has a high affinity for PC9 cells and facilitates its endocytosis. Compared with MP-SiO_2_ NP or MP-SiO_2_ NP loaded with EGFR targeting small compound GEF or MP-SiO_2_ NP capped with CET, the nano-medicine CET-capped GEF-loaded MP-SiO_2_ NP shows the best inhibitory efficacy in treatment of GEF-resistant PC9 cells *in vitro* and *in vivo*. Together, our findings show that GEF-loaded CET-capped nano-medicine is very efficient in overcoming the TKI resistance. Our design might serve as good method to specifically and effectively deliver the EGFR-TKIs and overcome drug resistance.

## Materials and Methods

### Synthesis of mesoporous SiO_2_ nanoparticles (MP-SiO_2_ NP)

For the synthesis of fluorescein-labeled MP-SiO_2_ NP, fluorescein isothiocyanate (FITC, 10 mg) was firstly reacted with 6 μl of (3-aminopropyl) triethoxysilane (APTES) in 1 ml ethanol overnight in the dark. Then, hexadecyltrimethylammonium bromide (CTAB, 0.80 g) was dissolved in 384 ml of double distilled water; sodium hydroxide (NaOH, 1 M, 5.6 ml) was added to CTAB solution, followed by adjusting the solution temperature to 80 °C. Subsequently, tetraethyl orthosilicate (TEOS, 4 mL) and dye-labeled APTES solution (400 μl) were added by dropwise during while stirring continued. The mixture was allowed to stir for 2 hours to give rise to white precipitates. Finally, the surfactant template, CTAB, was removed by refluxing in acidic ethanol solution (80 ml of ethanol and 1 ml of HCl) for 16 hours, and dried to give FITC-labeled MP-SiO_2_ NP. For the synthesis of mercapto-functionalized MP-SiO_2_ NP, the resulting MP-SiO_2_ NP (500 mg) was functionalized in the ethanol (50 ml) solution with 1 ml of 3-mercaptopropyltrimethoxysilane overnight. The particles were separated by centrifugation (8000 rpm, 10 minutes), washed several times with absolute ethanol, then with double distilled water, and dried in an oven at 65 °C overnight.

FITC was purchased from Tokyo Chemical Industry (TCI, Japan). (3-Aminopropyl) triethoxysilane (APTES), hexadecyltrimethy ammonium bromide (CTAB) and tetraethoxysilane (TEOS) were obtained from Sinopharm Chemical Reagent Co., Ltd. (Shanghai, China). 3-Mercaptopropyltriethoxysilane (MPTES) was purchased from Aladain (Shanghai, China). Sodium hydroxide (NaOH, 1 M) solution was obtained from Acros Organics (Belgium).

### Transmission electron microscopy (TEM) and N_2_ adsorption-desorption characterization

Transmission electron microscopy (TEM) image of the prepared MP-SiO_2_ NP was recorded using a JEM-1400 (JEOL, Japan) high resolution transmission electron microscope operating at 120 kV. N_2_ adsorption-desorption isotherms were performed on a Quadrasorb SI/MP (Quantachrome, America) automated sorption analyzer, the surface area and pore size distribution were determined using the Brunauer-Emmett-Teller (BET) and Barrett-Joyner-Halenda (BJH) analyses, respectively.

### Loading of mesoporous silica nano-medicine

A mixture consisting of monodispersed mercapto-functionalized MP-SiO_2_ NP solution was prepared by placing 10 mg MP-SiO_2_ NP in 1 ml of penicillin-streptomycin solution (5×) followed by the sonication of the mixture for 1 hour. The particles were collected by centrifugation (6000 rpm, 3 minutes), dissolved in 500 μl of DOX or GEF solution (2 mM) after the supernatant was removed, the suspension was stirred overnight at room temperature. Subsequently, 400 μl of CET (2.5 mg/ml), 110 μl of H_2_O_2_ (0.3%) and 50 μl of NaI (0.03 mg/ml) in HEPES buffer (20 mM, pH 7.0, containing 3 M NaCl) were added into the resulting solution and reacted for 2 hours^24^,^33^. Finally the mixture was washed at least six times with HEPES buffer (20 mM, pH 7.0) until no background color was observed.

Doxorubicin hydrochloride (DOX), hydrogen peroxide (H_2_O_2_), and sodium iodide (NaI) were purchased from Aladain (Shanghai, China). Gefitinib (GEF) was obtained from Sigma-Aldrich (America). CET was supported by Merck (Germany). BCA protein assay kit was obtained from Beyotime Biotechnology (Hunan, China). Three samples, (1) MP-SiO_2_ NP, (2) MP-SiO_2_ NP and CET, (3) MP-SiO_2_ NP and CET in the presence of 10 mM of H_2_O_2_ and 0.1 mM of NaI in HEPES buffer (20 mM, pH 7.0, containing 300 mM NaCl) were reacted for 2 hours. The particles were dispersed in 1 ml of HEPES buffer (20 mM, pH 7.0) after fully washed. Then the content of CET was determined using BCA protein assay kit. The modification of CET on the MP-SiO_2_ NP was estimated to be: (1) 0.5 μg/μl, (2) 1.1 μg/μl, and (3) 2.1 μg/μl. So the modification of CET is estimated to be 1.6 μg/μl.

### Release of mesoporous silica nano-medicine

The release of DOX from DOX-loaded MP-SiO_2_ NP capped with CET using the stimuli of GSH or DTT was described as follow. DOX-loaded MP-SiO_2_ NP, 10 mg, were introduced into 900 μl HEPES buffer (20 mM, pH 7.0), and the mixture was divided into five samples. Subsequently, 20 μl of aqueous solution of different concentrations of GSH or DTT were added to the samples that were allowed to react for a fixed time interval of 1 hour. The resulting mixtures were centrifuged, and the MP-SiO_2_ NP was separated. The absorbance spectra of the supernatant clear solutions were then recorded. For the time-dependent release of DOX from the system, a similar procedure was applied while subjecting the NP, for different time-intervals, at a fixed concentration of GSH or DTT (1 mM). The concentration of DOX in the release solution was evaluated by ultraviolet-visible spectra using an extinction coefficient of DOX (14700 L·mol^−1^·cm^−1^). The release amount of DOX in the system was estimated to 34.7 μM or 25.2 μM when using 1 mM GSH or 1 mM DTT as stimuli, respectively.

For using glutathione reduced ethyl ester (GSH-OEt) and diethyl maleate (DM) to regulate the concentration of GSH in cells, cells were plated on circular slides under the indicated conditions for 24 hours, subsequently 5 mM of GSH-OEt or 1 mM of DM were added to medium for 2 hours and washed once to remove the excess regulators. Then different concentrations of DOX-loaded nano-medicine without or with CET modification were incubated with cells for 24 hours, cells were crashed into suspension using cell disrupter after removing the unabsorbed nano-medicines. Finally, the absorbance spectrums of DOX were recorded on a ultraviolet-visible spectrophotometer at 490 nm. GSH-OEt and DM were purchased from Sigma and Aldrich, respectively.

### Ultraviolet-visible spectra

Ultraviolet-visible spectra were recorded on a Cary series ultraviolet-visible spectrophotometer (Agilent Technologies, America). The absorbance of DOX was measured at 490 nm. According to the definition of absorbance, we obtained the extinction coefficient of DOX is 14700L·mol^−1^·cm^−1^ using a calibration curve. Knowing the content of DOX present in the solution after the primary NP precipitation process, and knowing the amounts of DOX eliminated by the washing procedure, the loading amount of DOX is estimated to be about 650 μM.

### Cell culture

Beas2B and PC9 cells were bought from ATCC and cultured in Dulbecco’s modified Eagle’s medium (Hyclone, Thermo Scientific, USA) supplemented with 8% fetal bovine serum (FBS; Biochrom AG, Germany). PC9-DR cells were drug resistant cells derived from PC9 cells with continued GEF treatment. The cells were maintained at 37 °C in a humidified atmosphere with 5% CO_2_ and passaged every 2 days.

### Glutathione concentrition Test

The intracellular level of glutathione (GSH) was determined in 96 well black plate with a Synergy NEO multifunctional microplate reader (Bioteck, America) using a glutathione fluorometric assay kit (BioVision, America) as described in the instruction.

### Fluorescence scanning

Cells were plated on circular slides under the indicated conditions and the various indicated nanoparticles were added to medium 24 hours later. Cells were fixed with 4% polytetrafluoro ethylene for 10 min at room temperature and then counterstained with DAPI (Sigma) for 1 min. After mounted with anti-fade medium (Thermo Electron Corporation), the cells were imaged for FITC, DOX and DAPI with a fluorescence microscope (Nikon).

### MTT assay

To assess the effect of the modified nanoparticles, cells were plated in quadruplicate under the indicated conditions. After 24 hours, the various indicated nanoparticles were added to culture medium for 72 hours. Then the nanoparticles were withdrawn after 24 hours. Cells were stained with 3-(4,5-dimethyl-2-thiazolyl)-2,5-diphenyl-2-H-tetrazolium bromide (MTT) for 4 hours and assessed with Epoch multi-volume spectrophotometer system (570 nm/630 nm) on the fourth day.

### Western blot

Cells lysates were prepared and subjected to western blot analysis as previously reported^34^ with following primary antibodies: EGFR (A2909, ABclonal) and ACTIN (AB136452, Abcam).

### Immunohistochemical stainings

Tumors were fixed with 1 ml 4% paraformaldehyde (PFA) overnight, dehydrated in ethanol, embedded in paraffin and sectioned (5 μm). Slides were then deparaffinized in xylene and ethanol, and rehydrated in water. Antigen retrieval was performed by heating in a microwave for 30 minutes in sodium citrate buffer (pH 6.0). Slides were quenched in hydrogen peroxide (3%) to block endogenous peroxidase activity and then washed in TBST buffer. The primary antibodies were incubated at 4 °C overnight followed by using the SuperPicture™ Polymer Detection kit (Life Technologies) according to the manufacturer’s instructions as described^34^. Antibodies against the Ki67 (NCL-Ki67p, Leica Biosystems) and cleaved caspase 3 (9664, Cell Signaling) were used.

### *In Vivo* Treatment Studies

5 weeks male nude mice were injected subcutaneously with 5 × 10^6^ cells in the left and right flank, respectively. The mice were then randomly separated into different groups (in each group n > 6). When the cells form palpable tumors, animals were treated by intraperitoneal injection with free drugs dissolved in 5% Tween 80, 5% PEG 400, 90% water daily and treated by *in situ* injection with 50 μl modified MP-SiO_2_ NP daily (10 mg/ml). Tumor volume was calculated as follows: tumor size (mm^3^) = (longer measurement × shorter measurement^2^)/2. Tumor sizes were recorded every other day over the course of the studies. Mice were housed in a specific pathogen-free environment at the Shanghai Institute of Biochemistry and Cell Biology and treated in strict accordance with protocols approved by the Institutional Animal Care and Use Committee of the Shanghai Institutes for Biological Sciences, Chinese Academy of Sciences.

### Fluorescence activated Cell Sorting (FACS)

PC9 and Beas2B cells for FACS analyses were plated in triplicate. And 24 hours later, cells were treated with the indicated agents for 2 hours. Then, cells were collected and performed in the BD LSRII flow cytometry. For each test, at least 10000 cells were counted. The data were analyzed using FlowJo (Tree Star).

### Statistical Analysis

Statistical analyses were carried out using GraphPad Prism 5 software. Unless indicated, differences were compared using two-tailed Student’s t test.

## Additional Information

**How to cite this article**: Wang, Y. *et al*. Cetuximab-modified mesoporous silica nano-medicine specifically targets EGFR-mutant lung cancer and overcomes drug resistance. *Sci. Rep*. **6**, 25468; doi: 10.1038/srep25468 (2016).

## Supplementary Material

Supplementary Information
