# Supplementary material for: Cetuximab-modified mesoporous silica nano-medicine specifically targets EGFR-mutant lung cancer and overcomes drug resistance
Source: Sci Rep. 2016 May 6;6:25468. doi: 10.1038/srep25468 (PMC4858690; doi:10.1038/srep25468)
Supplement: Supplementary Information [file srep25468-s1.doc]

**Supplementary Information**

Cetuximab-modified mesoporous silica nano-medicine specifically targets EGFR-mutant lung cancer and overcomes drug resistance

Yuetong Wang^1,2,3^, Hsin-Yi Huang^1,2,3^, Liu Yang^1,2,3^, Zhanxia Zhang^1,2,3^*, Hongbin Ji^1,2,3,4^*

^1^Key Laboratory of Systems Biology,

^2^CAS center for Excellence in Molecular Cell Science,

^3^Innovation Center for Cell Signaling Network,

Institute of Biochemistry and Cell Biology, Shanghai Institutes for Biological Sciences, Chinese Academy of Science, Shanghai, 200031, China;

^4^School of Life Science and Technology, Shanghai Tech University, Shanghai, 200120, China.

^*^ Correspondence should be addressed to:

Dr. Hongbin Ji [hbji@sibcb.ac.cn](mailto:hbji@sibcb.ac.cn)

Dr. Zhanxia Zhang [zhanxia.zhang@sibcb.ac.cn](mailto:zhanxia.zhang@sibcb.ac.cn)

Keywords: mesoporous silica nano-medicine, drug resistance, EGFR-TKI, cetuximab

**Inventory of Supplemental Information**

Figure S1 is related to Figure 3

Figure S2 is related to Figure 5

Figure S3 is related to Figure 5

Supplementary Materials and Methods

Supplementary References

**
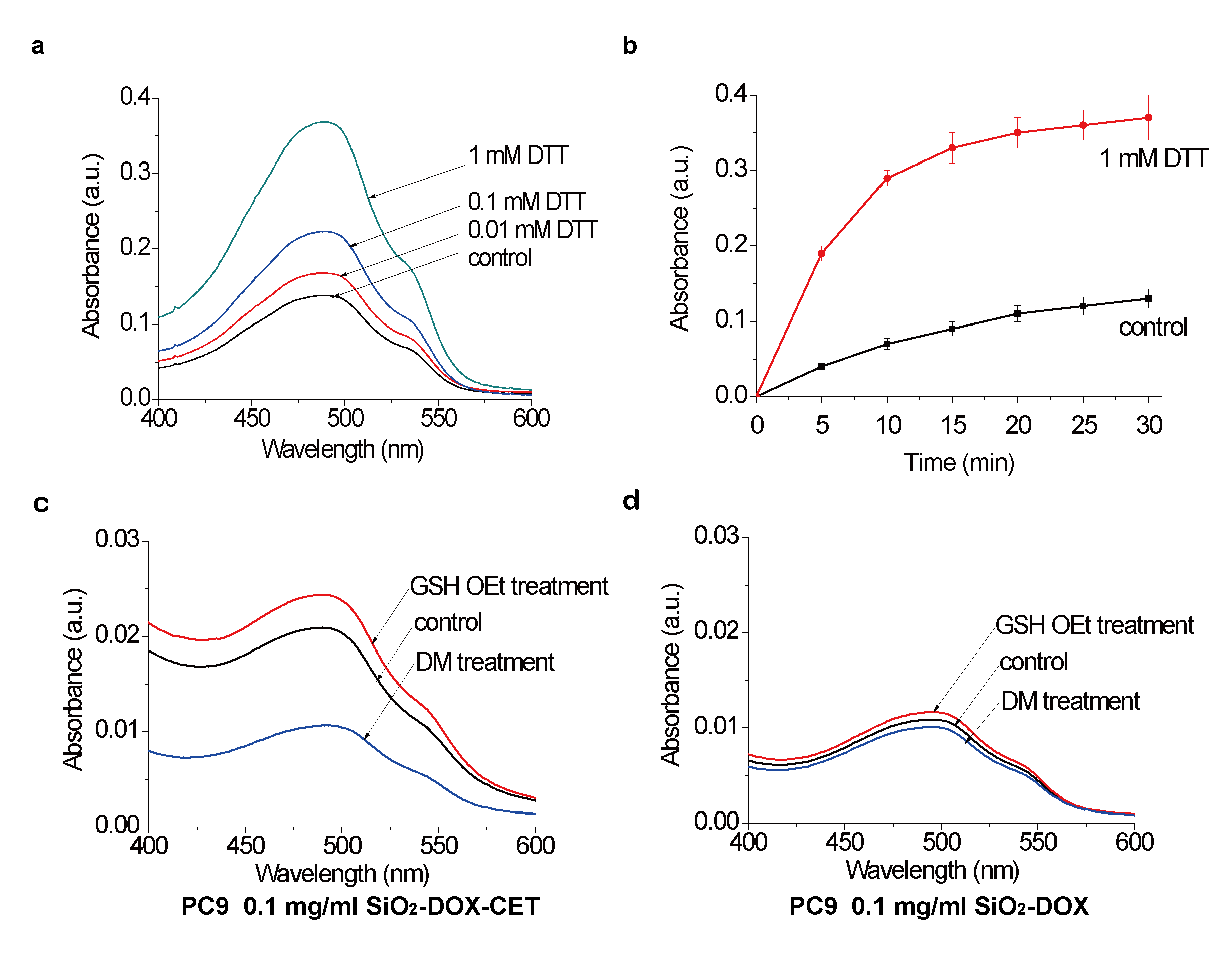
**

**Figure S1.** **Reduce or cleave the disulfide bonds triggered the release of loaded DOX from the CET-capped MP-SiO_2_ NP. (a)** Absorbance spectra corresponding to the released DOX upon subjecting MP-SiO_2_ NP (10 mg/ml) to different concentrations of DTT for 1 hour. **(b)** Time-dependent absorbance changes upon releasing DOX from the MP-SiO_2_ NP treat without or with 1 mM DTT. Bars represent mean ± SEM (n = 3). **(c)** Absorbance spectra corresponding to the released DOX upon subjecting the DOX-loaded CET-capped MP-SiO_2_ NP (0.1 mg/ml) with indicated treatment in PC9 cells. **(d)** The same as in (c) for the DOX-loaded MP-SiO_2_ NP sample.


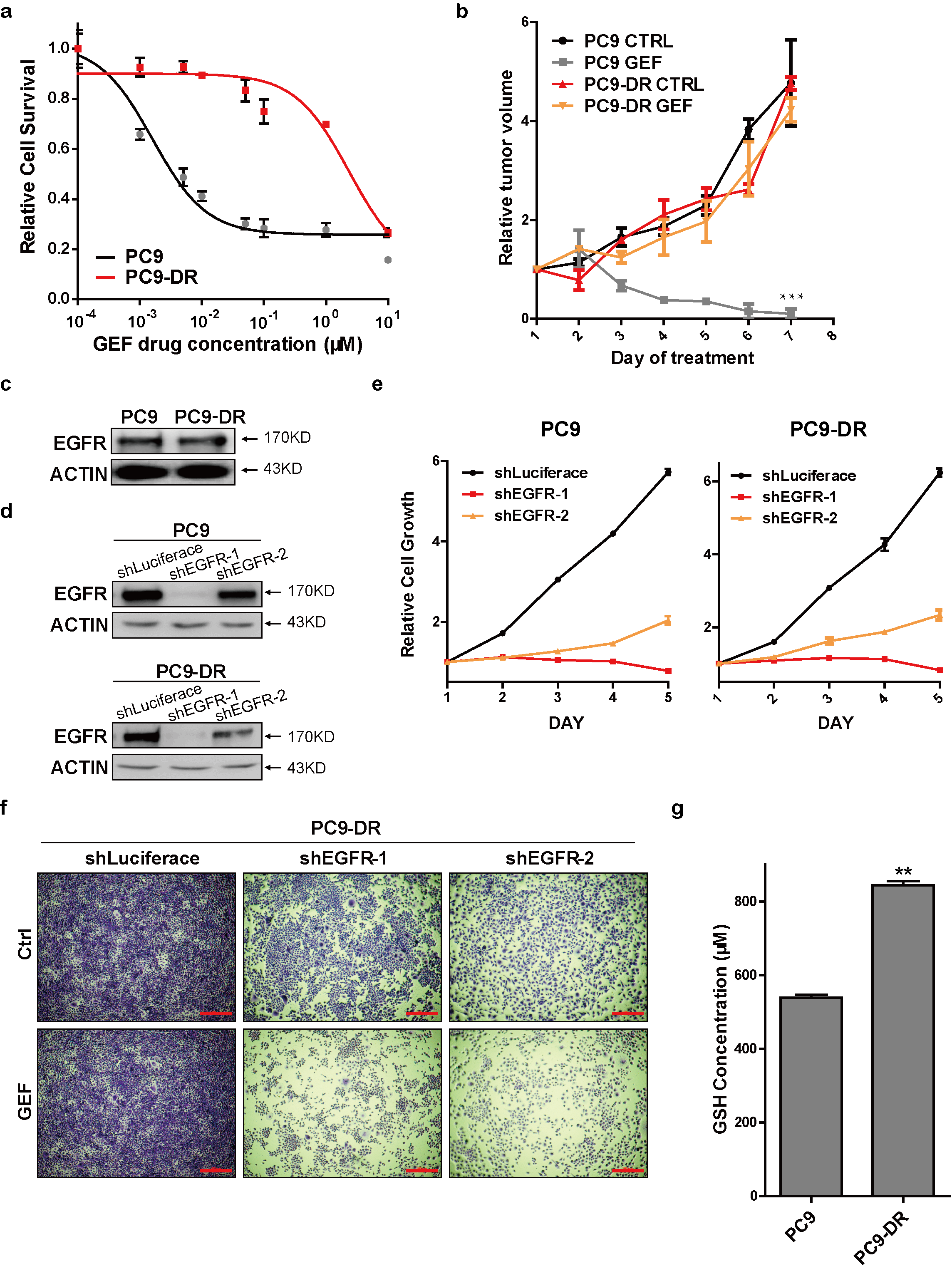


**Figure S2.** **PC9-DR cells are resistant to GEF treatment but still rely on EGFR signaling. (a)** Relative survival of PC9 and PC9-DR cells treated with different concentrations of GEF. Bars represent mean ± SEM (n = 4). **(b)** Relative tumor growth of PC9 and PC9-DR subcutaneous tumors treated with and without 0.3 mg/kg GEF daily. Bars represent mean ± SEM (n = 6). **(c)** Western blot analyses of EGFR in PC9 and PC9-DR cells. ACTIN served as internal control. **(d)** Western blot analyses of EGFR in PC9 and PC9-DR cells with or without EGFR knockdown. ACTIN served as internal control. **(e)** Growth curves of PC9 and PC9-DR cells with or without EGFR knockdown. Bars represent mean ± SEM (n = 6). **(e)** Growth curves of PC9 and PC9-DR cells with or without EGFR knockdown. Bars represent mean ± SEM (n = 6). **(f)** Crystal violet staining of PC9 and PC9-DR cells with or without EGFR knockdown. Scale bars: 500μm. **(g)** GSH levels of PC9 and PC9-DR cells. Bars represent mean ± SEM (n = 3). ** p < 0.01.


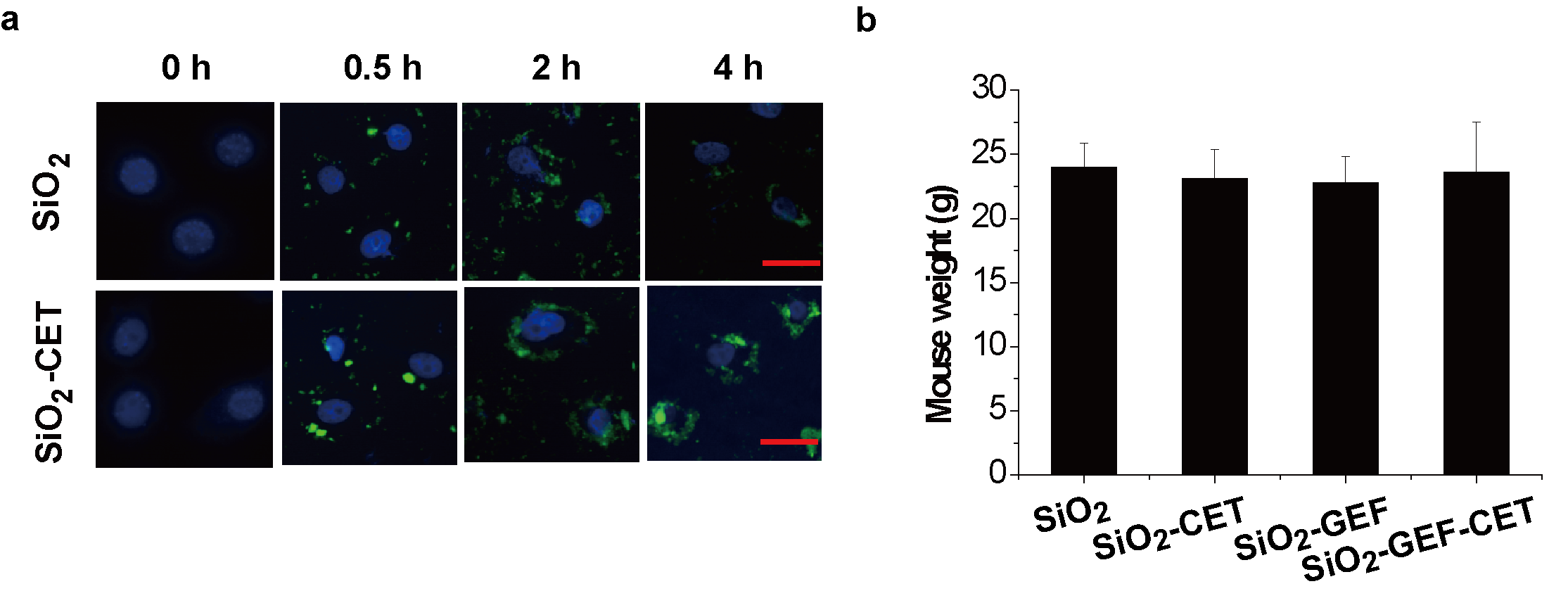


**Figure S3.** **CET-capped nanoparticles can overcome drug resistance of PC9-DR.** **(a)** Microscopic images of PC9-DR cells treated with 0.1 mg/ml MP-SiO_2_ NP or CET-capped MP-SiO_2_ NP at different time intervals (blue: DAPI; green: FITC). Scale bar: 50 μm. **(b)** Body weights of mice in different treatment groups before PC9-DR tumor collecting. Bars represent mean SEM (n = 6).
